# Supplementary material for: LipIDens: simulation assisted interpretation of lipid densities in cryo-EM structures of membrane proteins
Source: Nat Commun. 2023 Nov 27;14:7774. doi: 10.1038/s41467-023-43392-y (PMC10682427; doi:10.1038/s41467-023-43392-y)
Supplement: Supplementary file 1 — Supplementary Information [file 41467_2023_43392_MOESM1_ESM.pdf]

Supplementary information – LipIDens: Simulation assisted interpretation of lipid densities in cryo-EM structures of membrane proteins.

T. Bertie Ansell[1], Wanling Song[1,6], Claire E. Coupland[2,7], Loic Carrique[2], Robin A. Corey[1,8], Anna L. Duncan[1,9], C. Keith Cassidy[1,10], Maxwell M. G. Geurts[1], Tim Rasmussen[3], Andrew B. Ward[4], Christian Siebold[2], Phillip J. Stansfeld[5], Mark S. P. Sansom[1]

[1]Department of Biochemistry, University of Oxford, South Parks Road, Oxford, OX1 3QU, UK.
[2]Division of Structural Biology, Wellcome Centre for Human Genetics, University of Oxford, Roosevelt Drive, Oxford, OX3 7BN, UK.
[3]Biocenter and Rudolf-Virchow-Zentrum, Universität Würzburg, Haus D15, Josef-Schneider-Str. 2, 97080 Würzburg, Germany
[4]Department of Integrative Structural and Computational Biology, The Scripps Research Institute, La Jolla, California 92037, USA
[5]School of Life Sciences & Department of Chemistry, University of Warwick, Coventry, CV4 7AL, UK.
[6]MSD R&D Innovation Centre, 120 Moorgate, London, EC2M 6UR, UK.
[7]Molecular Medicine Program, The Hospital for Sick Children, Toronto, M5G 0A4, Canada.
[8]School of Physiology, Pharmacology and Neuroscience, University of Bristol, Bristol, BS8 1TD, UK.
[9]Department of Chemistry, Aarhus University, Lagelsandsgade 140, 8000 Aarhus C, Denmark.
[10]Department of Physics and Astronomy, University of Missouri-Columbia, Columbia, MO 65211, USA.

Supplementary Information:

Best practices for users and pipeline limitations:

- When running the LipIDens pipeline using the master python file (`'lipidens_master_run.py'`) default variables and parameters are provided. To accept these parameters press the ENTER/RETURN key. In addition, all steps of the LipIDens pipeline are described in detail in the accompanying LipIDens protocol.
- For smooth running we recommend users are familiar with setting up and performing MD simulations using GROMACS.
- We recommend using the `lipidens_master_run.py` file for extended running of the pipeline. However, the jupyter notebook is useful for tutorial processes.
- LipIDens will provide the most likely identity of lipids bound to a site under a given lipid composition. The pipeline cannot be used to definitively identify lipid-like densities due to the plethora (>1000s) of lipids present within cellular membranes. Hence users should select bilayer conditions which provide a suitable minimal mimetic of the native membrane or experimental conditions.
- Cellular membranes contain thousands of lipid species, a subset of which have accompanying CG parameters files, hence users should check the availability of specific lipid parameters for a particular forcefield. In particular, it may be necessary to approximate tail lengths and saturation to the nearest available lipid.
- The LipIDens pipeline will print out warnings when appropriate and terminate the protocol when GROMACS errors occur during simulation setups. The output of GROMACS commands are provided within the '*output_files'* directory within each simulation replicate. If errors occur, please review these files and fix the underlying problem (e.g. missing atoms in the .pdb file) before rerunning the code.
- We recommend a CG simulation time of at least 10 μs and to run multiple repeat simulations (at least 8). If convergence of lipid interactions is not reached during the simulation timeframe, then simulations should be extended. The '*Screening PyLipID section*' of the accompanying protocol details best practices for assessing convergence of kinetic parameters.
- It is good practice to test PyLipID cut-offs for at least one phospholipid and any other lipids/sterols with significantly different molecular structures e.g. cholesterol or cardiolipin.
- When running the analysis stages of the pipeline the '`stride`' variable can be used to skip X number of frames. This is useful for dealing with slower run times or computational memory errors during PyLipID analysis.
- LipIDens will provide warnings when binding sites are assigned multiple times to the site comparison dictionary (`BindingSite_ID_dict`). Users should check these sites carefully, remove any poorly defined sites and/or additional site occurrences.
- While LipIDens can facilitate modelling into structures with lipid-like densities it cannot predict the functional consequence of bound lipids. Hence, the biological relevance of bound lipids should be assessed by accompanying biochemical and/or spectral analyses.

Supplementary Figures:

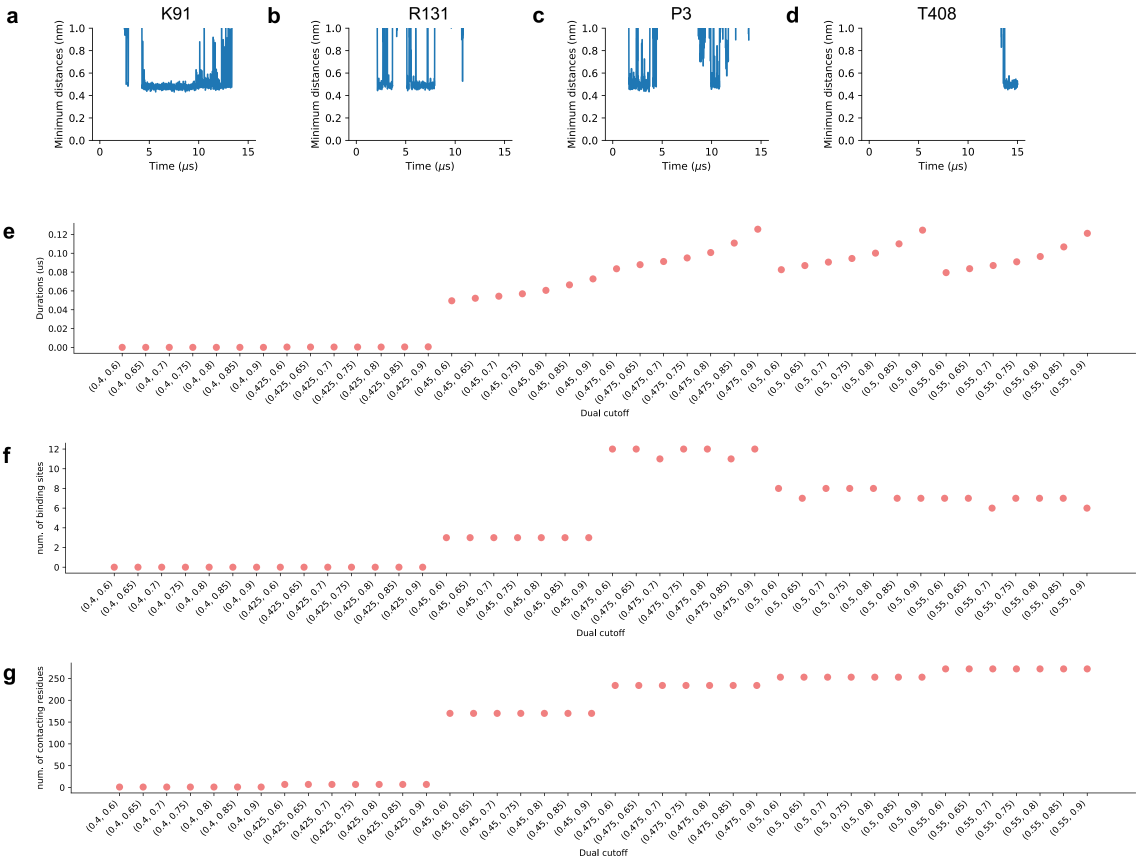

Supplementary Figure 1: Tuning PyLipID cut-off values: interactions of HHAT with PIP_2

Plotted outputs from PyLipID cut-off testing. **a-d**) Minimum distances between HHAT residues **a**) K91 **b**) R131 **c**) P3 and **d**) T408 and a PIP_2 molecule across one 15 μs CG simulation. The minimum distance was calculated between any bead of the residue and any bead of the lipid. For clarity, only those interactions which came within 0.65 nm (`distance_threshold`) for at least 30 frames (`contact_frames`) of the simulation are plotted. **e-g**) Exhaustive testing of a range of lower and upper cut-off combinations for HHAT-PIP_2 interactions (n=10 x 15 μs CG independent simulations). Plots show the effect of the selected cut-offs on **e**) interaction duration times **f**) the number of calculated binding sites and **g**) the number of interacting residues.

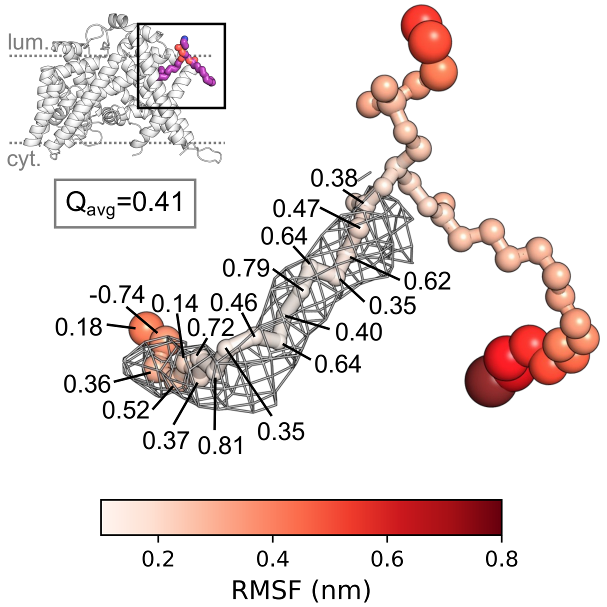

Supplementary Figure 2: Comparison of lipid fluctuation with the cryo-EM density.
The per atom root mean square fluctuation (RMSF) of a POPE lipid bound to HHAT (boxed) across n=5 x 200 ns independent atomistic simulations. POPE atom spheres are scaled by RMSF value and coloured from low (white) to high (red). The per atom Q score[1] was used to assess how well the simulation derived lipid pose matched the cryo-EM density.

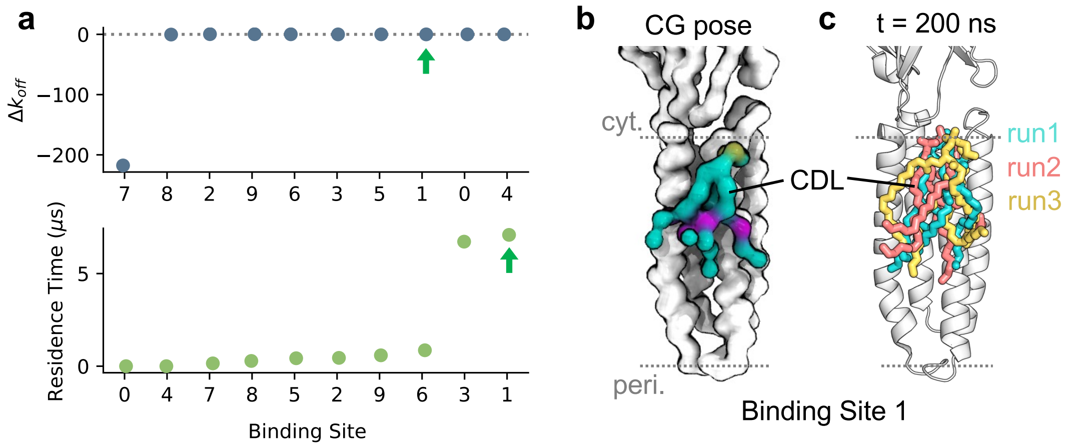

Supplementary Figure 3: Cardiolipin binding to ELIC.
a) Cardiolipin (CDL) binding sites (BS) ranked from worst to best Δk_{off} (Δk_{off} = k_{off} from curve fitting – bootstrapped k_{off}) or lowest to highest residence time (n=10 x 15 μs independent CG simulations). The CDL binding site with the longest residence time, BS1, is arrowed. **b)** Top ranked CG binding pose for CDL at BS1. **c)** Snapshots of the CDL binding pose at the end of n=3 x 200 ns independent atomistic simulations initiated using the CG CDL binding pose in **b**.

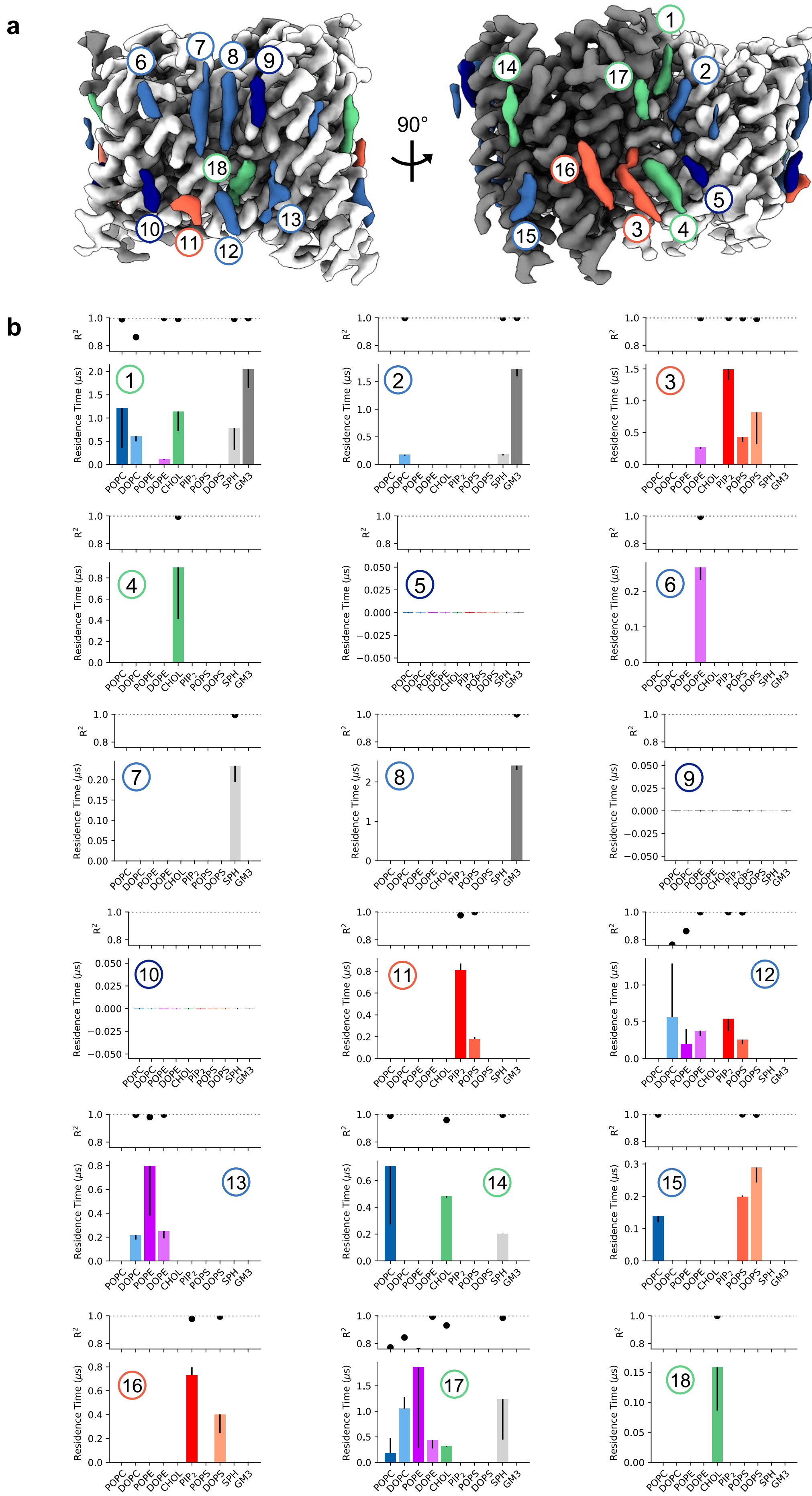
a
90°
1
2
3
4
5
6
7
8
9
10
11
12
13
14
15
16
17
18
b
R²
Residence Time (μs)
POPC
DOPC
POPE
DOPE
CHOL
PIP₂
POPS
DOPS
SPH
GM3

Supplementary Figure 4: Interpretation of lipid densities surrounding OTOP1.
a) Numbered lipid-like densities surrounding OTOP1, coloured as in Fig. 5. **b)** Residence time of lipids bound at sites corresponding to each numbered density, where the k_{off} was derived from bi-exponential curve fitting of the interaction survival function. Asymmetric error bars indicate a second k_{off} value obtained via bootstrapping to the same data. Lipid binding sites and residence times were calculated using PyLipID and a 0.475/0.7 nm cut-off from n=10 x 15 μs independent CG simulations of OTOP1.

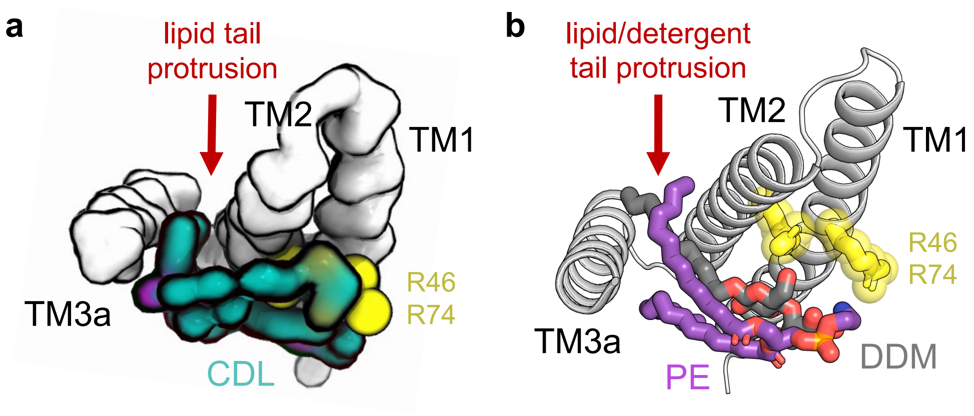

Supplementary Figure 5: Lipid/detergent tail protrusion between TM helices of MscS.
a) Cardiolipin (CDL) binding pose from CG simulations of MscS (viewed from the cytosolic membrane). The transmembrane helices of one monomer of MscS are shown as white surface and the position of residues coordinating the CDL headgroup are shown as yellow spheres. CDL tail protrusion between TM2/TM3a is arrowed. **b)** Structure of MscS (PDBid 7ONJ)[2] in cartoon representation. The position of bound lipid (PE, purple) and detergent (DDM, grey) tails between TM2/TM3a is arrowed. Headgroup coordinating residues are shown as yellow sticks.

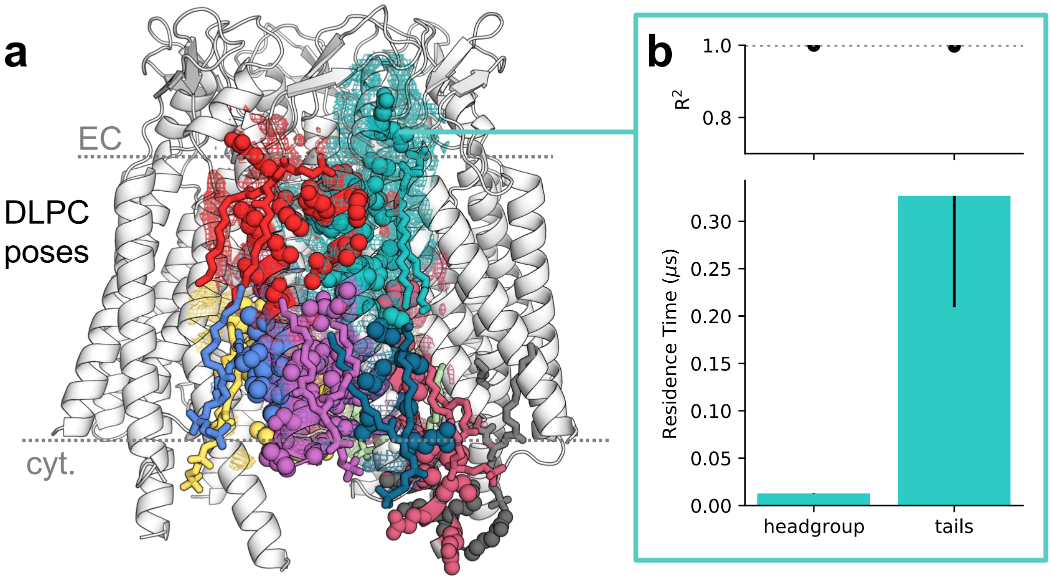

Supplementary Figure 6: DLPC binding sites on Connexin-50.
a) Snapshot from the interactive PyMOL session for Connexin-50, comparing DLPC binding poses with partitioned site densities. DLPC molecules are shown as sticks overlayed with site densities (mesh) and coloured accordingly for each binding site. **b)** Contribution of DLPC headgroup *vs.* tails to residence times for the extracellular (EC) binding site shown in cyan (n=10 x 15 μs independent CG simulations). Residence times were derived from k_{off} values obtained by bi-exponential curve fitting of the interaction survival function. Asymmetric error bars indicate a second k_{off} value obtained via bootstrapping to the same data.

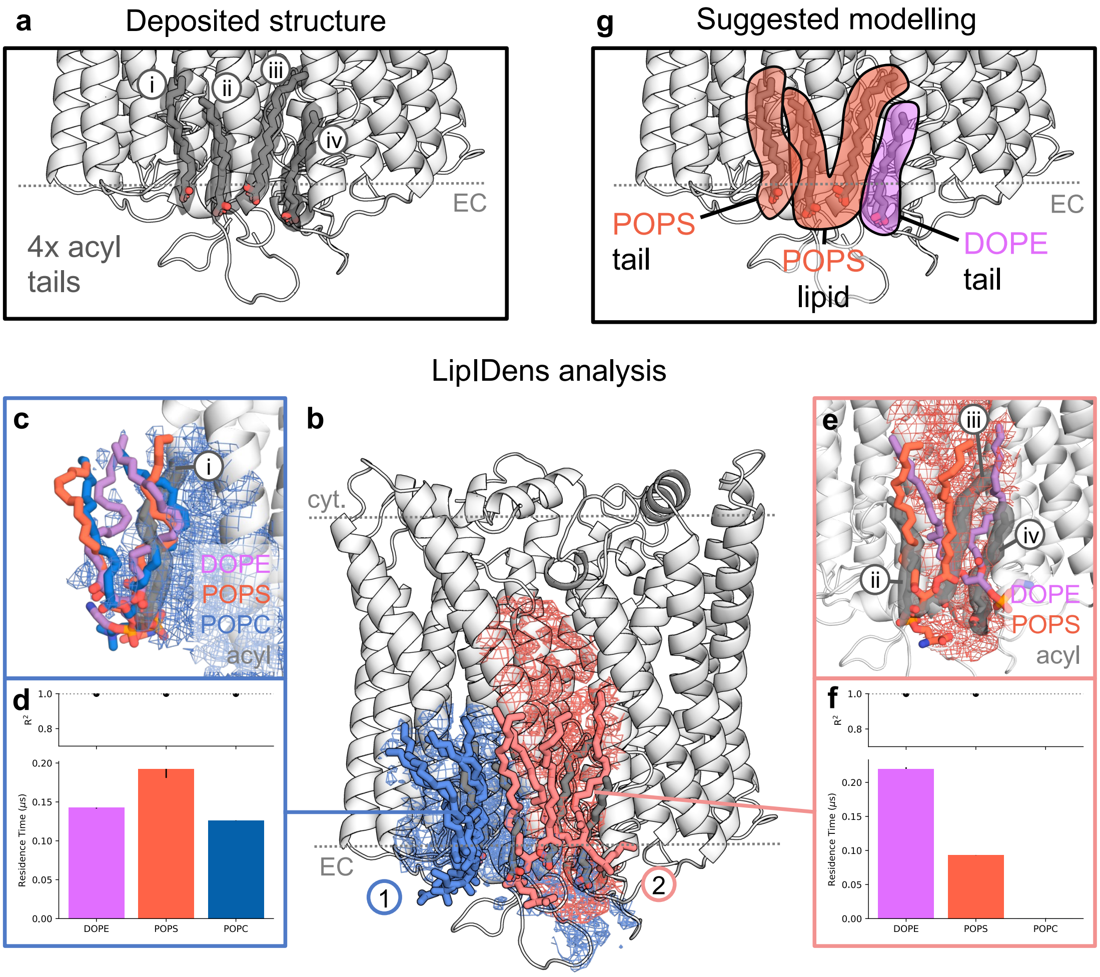

Supplementary Figure 7: Relative contribution of distinct lipids to binding sites on ChRmine.

a) Position of four acyl-tails (grey sticks, numbered i-iv) modelled into lipid-like densities (grey surface) within the extracellular (EC) leaflet of the ChRmine structure (PDBid: 7SFK)[3]. **b)** Overlay of lipid binding poses for DOPE, POPE and POPS (sticks) to BS1 (blue) and BS2 (red) with partitioned site densities (mesh). **c)** Comparison of top-ranked lipid binding poses at BS1 with density-i showing co-localisation of a single lipid tail while the second tail faces the surrounding membrane. Poses correspond to those directly backmapped from n=10 x 15 μs independent CG simulations (without refinement using atomistic simulations). **d)** Relative residence times for lipids bound to BS1, where the k_{off} was derived from bi-exponential curve fitting of the interaction survival function. Asymmetric error bars indicate a second k_{off} value obtained via bootstrapping to the same data. **e)** Overlay of DOPE and POPS poses with densities-ii to -iv at BS2. **f)** Relative residence time plot for BS2 (plotted as in **d**). **g)** Suggested lipid modelling based upon analysis of top ranked lipid binding poses, relative residence time plots and density connectivity. We suggest the most likely identity of density-i and density-iv are a single tail of POPS and DOPE respectively while densities-ii/iii correspond to a single POPS lipid.

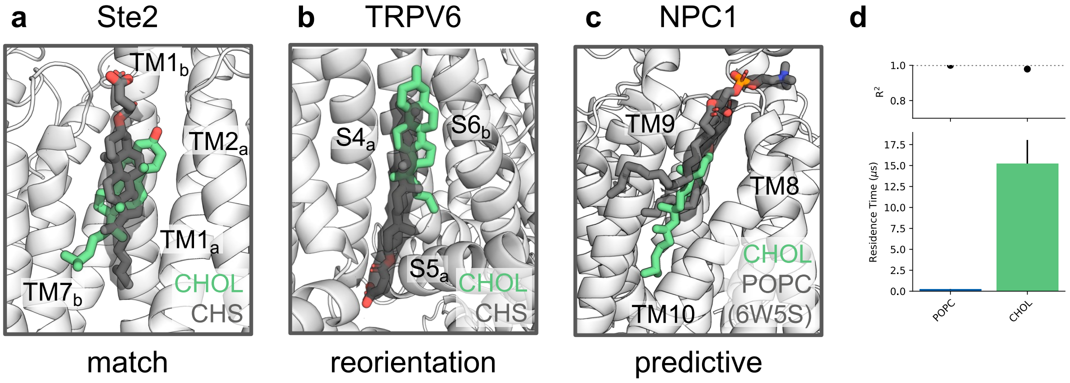

Supplementary Figure 8: Comparison of cholesterol and CHS binding sites. Overlay of the top-ranked cholesterol binding pose (green) with modelled CHS moieties (grey) and densities (grey surface) for **a)** the dimeric interface of Ste2 (Supplementary Fig. 9, BS1) and **b)** a pore proximal cavity on TRPV6 (Fig. 6, BS13). **c)** Cholesterol binding site with the longest residence time on NPC1 between TM8-12, overlayed with a modelled POPC density (grey) from the cryo-EM map of a different NPC1 structure (PDBid: 6W5S)[4]. **d)** Relative residence time for POPC and cholesterol bound to the site on NPC1 shown in **c**. Residence times were derived from k_{off} values obtained from bi-exponential curve fitting of the interaction survival function. Asymmetric error bars indicate a second k_{off} value obtained via bootstrapping to the same data. Poses correspond to those directly backmapped from CG resolution (n=10 x 15 μs independent CG simulations of Ste2 and TRPV6 and n=10 x 30 μs for NPC1).

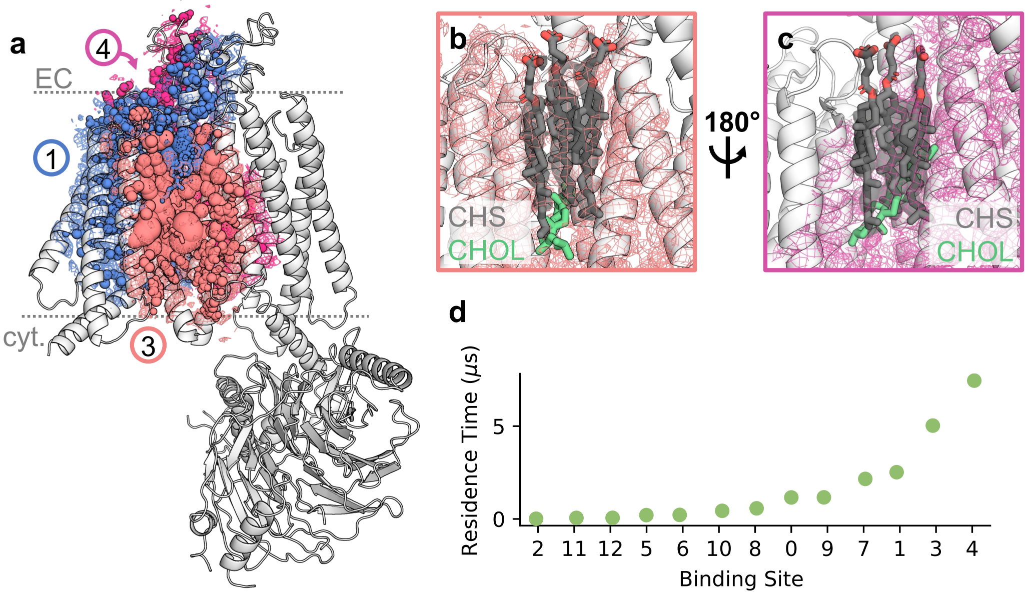

Supplementary Figure 9: Cholesterol binding sites on Ste2.
a) Top three cholesterol binding sites on Ste2 with the longest residence times (BS1, BS3, BS4). Residue side chains comprising each site are shown as spheres scaled by residence time, cholesterol is shown as sticks and partitioned site densities are shown as mesh. Comparison of the top-ranked cholesterol pose for **b)** BS3 and **c)** BS4 at the Ste2 dimeric interface with modelled CHS molecules (grey) and associated densities (grey surface). **d)** Ranked residence times for all cholesterol binding sites on Ste2 across n=10 x 15 μs independent CG simulations.

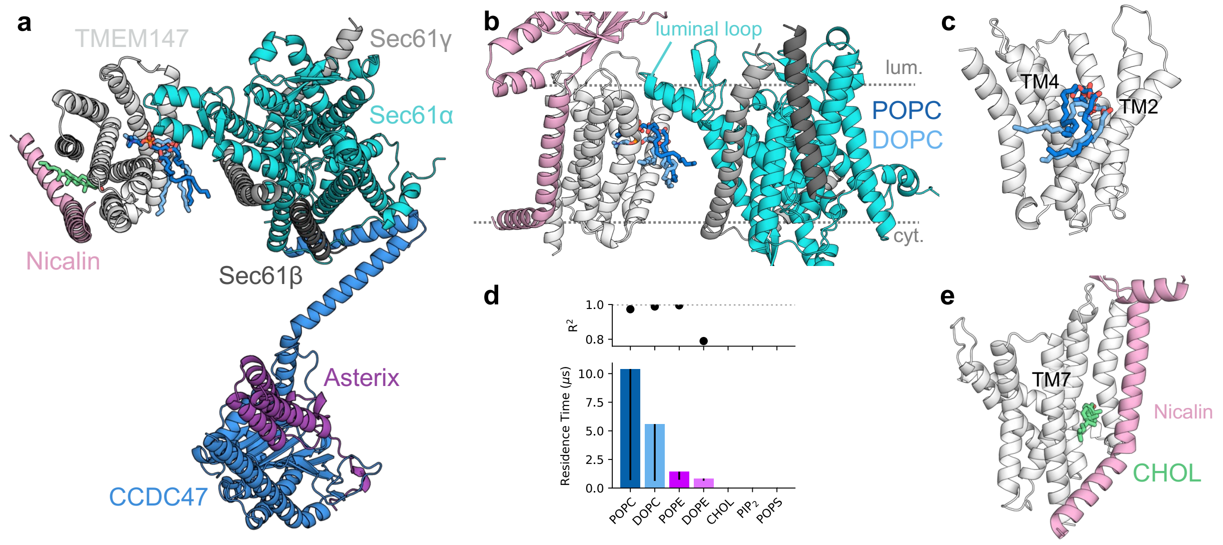

Supplementary Fig. 10: Predicted lipid binding sites on the PAT-Sec61 translocon complex.

a) Structure of the PAT-Sec61 complex as viewed from the ER luminal surface (PDBid: 7TM3)[5]. Seven proteins within the complex (CCDC47, Asterix, Sec61α, Sec61β, Sec61γ, TMEM147 and Nicalin) were used in simulations and are individually coloured. The ribosome was excluded from simulations. For clarity only the transmembrane region of Nicalin is shown. Lipids bound to two binding sites on TMEM147 are shown as sticks. **b-c)** Predicted POPC/DOPC binding site on TMEM147 sandwiched between TMEM147 TM2/TM4 and a luminal loop of Sec61α across n=10 x 15 μs independent CG simulations. **d)** Relative residence time of lipids bound to the site shown in **b/c**. Residence times were derived from k_{off} values obtained from bi-exponential curve fitting of the interaction survival function. Asymmetric error bars indicate a second k_{off} value obtained via bootstrapping to the same data. **e)** Predicted location of a cholesterol binding site between TMEM147 TM7 and the transmembrane helix of Nicalin.

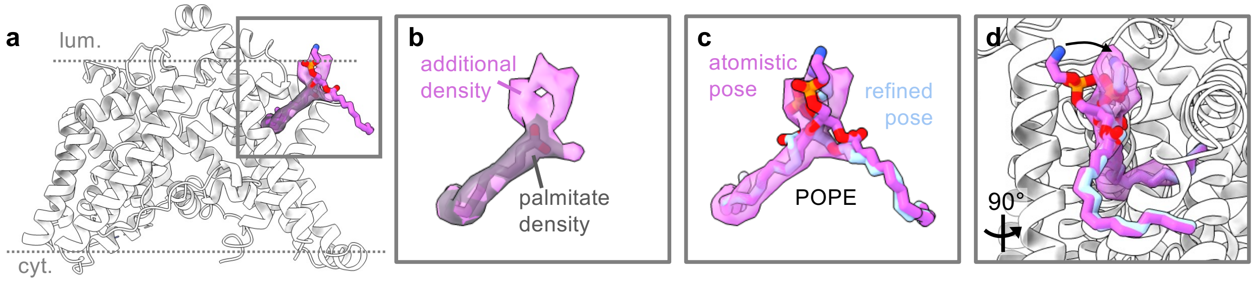

Supplementary Fig. 11: Modelling and refinement of POPE bound to HHAT.
a-b) Comparison of density assigned to a palmitate chain (grey) with a POPE pose after refinement using atomistic simulations (purple) bound between the N- and C-terminal helices of HHAT (PDBid: 7Q1U). Additional density not accounted for by protein side-chains, and unassigned within the deposited structure is shown as purple surface at the approximate position of lipid headgroups. **c)** Overlay of POPE poses after atomistic simulation (purple sticks) and additional fitting to the cryo-EM density (light blue sticks). **d)** The phosphate and ethanolamine chemical groups of the refined POPE headgroup (blue sticks) position within the additional density (purple). Arrows indicate headgroup movement compared to the pose from atomistic simulations (purple sticks).

Supplementary Tables:

Supplementary Table 1: Lipid compositions used in CG simulations.

Protein	Bilayer choice	CG bilayer composition
HHAT	Endoplasmic reticulum	Upper: POPC (35%), DOPC (35%), POPE (8%), DOPE (7%), cholesterol (10%), palmitate (PCN) (5%)<br>Lower: POPC (15%), DOPC (15%), POPE (19%), DOPE (18%), POPS (8%), PIP_2 (10%), cholesterol (10%), palmitate (PCN) (5%)
OTOP1	Plasma membrane	Upper: POPC (15%), DOPC (15%), POPE (2.5%), DOPE (2.5%), sphingomyelin (DPSM) (22%), GM3 (10%), cholesterol (33%)<br>Lower: POPC (7.5%), DOPC (7.5%), POPE (15%), DOPE (15%), POPS (7.5%), DOPS (7.5%), PIP_2 (7%), cholesterol (33%)
ELIC	*E. coli* inner membrane	POPE (67%), POPG (23%), cardiolipin (CDL2) (10%)
MscS	*E. coli* inner membrane	POPE (67%), POPG (23%), cardiolipin (CDL2) (10%)
Ste2	Simple	POPC (70%), cholesterol (30%)
Connexin-50	Nanodisc composition mimetic	DLPC (100%)
ChRmine	Nanodisc composition	DOPE (50%), POPS (25%), POPC (25%)
TRPV6	Plasma membrane	Upper: POPC (20%), DOPC (20%), POPE (5%), DOPE (5%), sphingomyelin (DPSM) (15%), GM3 (10%), cholesterol (25%)<br>Lower: POPC (5%), DOPC (5%), POPE (20%), DOPE (20%), POPS (8%), DOPS (7%), PIP_2 (10%), cholesterol (25%)
NPC1	Binary mixture	POPC (90%), cholesterol (10%)
PAT-Sec61 complex	ER membrane	Upper: POPC (37%), DOPC (37%), POPE (8%), DOPE (8%), cholesterol (10%)<br>Lower: POPC (15%), DOPC (15%), POPE (20%), DOPE (20%), POPS (10%), PIP_2 (10%), cholesterol (10%)

Supplementary Table 2: Predefined lipid compositions.

Predefined bilayer type	CG bilayer composition (`insane.py` format)[6]
Simple	-u POPC:70 -u CHOL:30 -l POPC:70 -l CHOL:30
Plasma membrane	-u POPC:20 -u DOPC:20 -u POPE:5 -u DOPE:5 -u DPSM:15 -u DPG3:10 -u CHOL:25 -l POPC:5 -l DOPC:5 -l POPE:20 -l DOPE:20 -l POPS:8 -l DOPS:7 -l POP2:10 -l CHOL:25
ER membrane	-u POPC:37 -u DOPC:37 -u POPE:8 -u DOPE:8 -u CHOL:10 -l POPC:15 -l DOPC:15 -l POPE:20 -l DOPE:20 -l POPS:10 -l POP2:10 -l CHOL:10
Raft-like microdomain	-u DPPC:27 -u DPPE:8 -u DPSM:15 -u DPG3:10 -u CHOL:40 -l DPPC:15 -l DPPE:35 -l DPPS:10 -l CHOL:40
Gram neg. inner membrane	-u POPE:67 -u POPG:23 -u CDL2:10 -l POPE:67 -l POPG:23 -l CDL2:10
Gram neg. outer membrane	-u PGIN:100 -l POPE:90 -l POPG:5 -l CDL2:5

References:

1. Pintilie, G. *et al.* Measurement of atom resolvability in cryo-EM maps with Q-scores. *Nat. Methods* **17**, 328–334 (2020).
2. Flegler, V. J. *et al.* Mechanosensitive channel gating by delipidation. *Proc. Natl. Acad. Sci. U. S. A.* **118**, 1–8 (2021).
3. Tucker, K., Sridharan, S., Adesnik, H. & Brohawn, S. G. Cryo-EM structures of the channelrhodopsin ChRmine in lipid nanodiscs. *Nat. Commun.* **13**, 1–12 (2022).
4. Qian, H. *et al.* Structural Basis of Low-pH-Dependent Lysosomal Cholesterol Egress by NPC1 and NPC2. *Cell* **182**, 98-111.e18 (2020).
5. Smalinskaitė, L., Kim, M. K., Lewis, A. J. O., Keenan, R. J. & Hegde, R. S. Mechanism of an intramembrane chaperone for multipass membrane proteins. *Nature* **611**, 161–166 (2022).
6. Wassenaar, T. A., Ingólfsson, H. I., Böckmann, R. A., Tieleman, D. P. & Marrink, S. J. Computational Lipidomics with *insane* : A Versatile Tool for Generating Custom Membranes for Molecular Simulations. *J. Chem. Theory Comput.* **11**, 2144–2155 (2015).
